# Could BCG Vaccination Induce Protective Trained Immunity for SARS-CoV-2?

**DOI:** 10.3389/fimmu.2020.00970

**Published:** 2020-05-08

**Authors:** Camila Covián, Angello Retamal-Díaz, Susan M. Bueno, Alexis M. Kalergis

**Affiliations:** ^1^Millenium Institute on Immunology and Immunotherapy, Departamento de Genética Molecular y Microbiología, Facultad de Ciencias Biológicas, Pontificia Universidad Católica de Chile, Santiago, Chile; ^2^Millennium Institute on Immunology and Immunotherapy, Departamento de Endocrinología, Facultad de Medicina, Escuela de Medicina, Pontificia Universidad Católica de Chile, Santiago, Chile

**Keywords:** SARS-CoV-2, COVID-19, BCG, innate immunity, trained immunity, vaccine

## Abstract

Trained immunity is a type of non-specific memory-like immune response induced by some pathogens and vaccines, such as BCG, which can confer antigen-independent protection against a wide variety of pathogens. The BCG vaccine has been extensively used to protect against tuberculosis for almost a 100 years. Interestingly, this vaccine reduces children's mortality caused by infections unrelated to *Mycobacterium tuberculosis* infection, a phenomenon thought to be due to the induction of trained immunity. The SARS-CoV-2 pandemic has infected, as of April 22, 2020, 2,623,231 people globally, causing a major public health problem worldwide. Currently, no vaccine or treatment is available to control this pandemic. We analyzed the number of positive cases and deaths in different countries and correlated them with the inclusion of BCG vaccination at birth in their national vaccination programs. Interestingly, those countries where BCG vaccination is given at birth have shown a lower contagion rate and fewer COVID-19-related deaths, suggesting that this vaccine may induce trained immunity that could confer some protection for SARS-CoV-2.

## Introduction

Vaccines are considered one of the most important public health achievements of science and medicine, saving the lives of millions of people as well as being one of the most impactful measures against preventing disease (1, 2).

Vaccines stimulate the activation of the adaptive immune response and the development of immunological memory, consisting of antigen-specific T and B cells that protect against infections by pathogens (3, 4). For the development of a vaccine, it is necessary to know the structure of the pathogen against which the formulation is designed, as well as the immunogenic components, such as adjuvants. However, the development of a new formulation and pre-clinical and clinical assays can take a significant amount of time (5). Considering the urgency around improving the immune response of the population when confronting a rapidly disseminating pandemic disease, such as the one caused by severe acute respiratory syndrome coronavirus (SARS-CoV-2), there is little or no capacity to develop a new formulation to immunize the population and comply with all the required regulatory steps. Therefore, strategies to steer the host immune system to adequately defend itself from a new viral infection, such as SARS-CoV-2, are required. A potential approach to achieve this goal consists of inducing trained immunity in the individual, which has been shown to enhance protection against some viruses, such as yellow fever virus (6). The concept of trained immunity refers to an increased immune response to an unrelated infection mediated by the innate immune system, specifically by monocytes, macrophages, and NK cells (7). This type of immune response is non-specific, can be either to the same or different microorganisms, and is independent of T and B cell responses (8).

**Figure 1:**
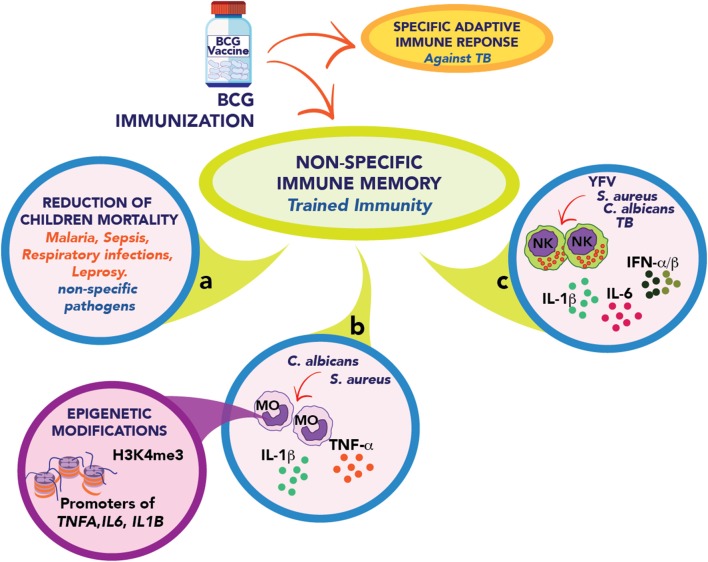
Schematic representation of trained immunity elicits by BCG immunization. **(a)** The BCG vaccine develops a specific adaptive and protective immune response against *M. tuberculosis*. It also promotes a non-specific immune memory called Trained immunity. The BCG vaccine contributes in many countries to reducing the infection rate of children against other unrelated pathogens such as malaria, respiratory infections, and leprosy. **(b)** BCG vaccination in adults leads to a trained phenotype in circulating monocytes (MO) that quickly respond, secreting IL-1β, TNF-α, and IL-6 after stimulation with unrelated pathogens such as *S. aureus* and *C. albicans*. This response is explained by epigenetic modifications in regulatory elements of *tnfa, il6*, and *il1b* genes. **(c)** In healthy human volunteers, the vaccination enhanced the capacity of NK cells to secrete proinflammatory cytokines and type I interferons after stimulation with *M. tuberculosis, S. aureus, C. albicans*, and Yellow fever virus (YFV).

The most prominent example for the induction of trained immunity is the *Bacillus* Calmette-Guérin (BCG), the only licensed vaccine against tuberculosis, a live attenuated vaccine that has widely been used in humans for almost a 100 years (9). Besides protection against tuberculosis, BCG has been shown to reduce the mortality of children due to infection by unrelated pathogens due to a non-specific immune cross-protection (Figure 1) (10, 11). In recent years, it has been shown that this effect is a consequence of the type of non-specific immune memory induced after vaccination as part of protective “trained immunity” (7). This type of immunological memory is developed by innate immune cells, such as monocytes, macrophages, and natural killer (NK) cells (12, 13), and can be efficiently induced by BCG (13–15), β-glucan (16), or *Candida albicans* (17). The “trained” state allows the cell to respond in a faster and stronger way against several microbial infections (13, 14).

Although BCG can induce the development of trained immunity, this does not imply that infection or the disease caused by *M. tuberculosis* may have the same response. BCG is an attenuated strain of *M. bovis* obtained after 230 culture passages with different genome deletions (18, 19). These deletions alter the expression of different *Mycobacterium* virulence factors (18–21). The differential expression of these molecules leads to the induction of different immune responses when exposed to an *M. tuberculosis* infection or BCG vaccination (8, 22). Trained immunity induction has only been described for BCG vaccination (13–15, 23).

Trained immunity has been shown to confer protection against a wide variety of pathogens, including bacteria (24), fungi (13), viruses (6), and protozoa (16). After the induction of trained immunity in mice, it protects against infections from *Escherichia coli, Listeria monocytogenes, Staphylococcus aureus, Citrobacter rodentium*, and *Pseudomonas aeruginosa* (24). In humans, trained monocytes have shown to increase production of IL-1β, TNF-α, and IFN-γ when stimulated with *Mycobacterium tuberculosis, S. aureus*, and *C. albicans* (13). In an experimental model of yellow fever viral infection, the induction of trained immunity reduces the levels of viremia (6). Interestingly, IL-1β plays a crucial role in mediating this innate response (6). In mice, the induction of trained immunity can protect in a model of *Leishmania braziliensis* infection (16). Furthermore, the BCG vaccination has been shown to be effective at preventing acute upper respiratory tract infections in the elderly (25) and is associated with reduced asthma and atopy in adults (26). Although intravenous BCG administration fails to protect against experimental influenza in mice (27), the effectiveness of the cross-protection induced by this vaccine varies depending on the route of administration (27–29). In fact, intraperitoneal and intranasal administration of this vaccine was able to protect against influenza infection, with the intranasal route being more effective (28, 29).

This antigen-unspecific immune “memory” induced by trained innate immune cells can last for up to 3 months post-vaccination (13). Such an effect in the innate immune system is lost 1 year after vaccination, with IL-1β and TNF-α production levels comparable to non-trained cells after *in vitro* stimulation with *C. albicans* or *S. aureus* (15). Based on the fact that trained immunity is a non-specific immunological memory which is rapidly developed and lasts a limited time, this suggests that trained immunity represents a good tool to induce non-specific protection against pathogens when a specific vaccine is not available, for example in a pandemic pathogen scenario. Despite its short duration, the exposure to a pathogen when trained immunity is present is thought to steer the endogenous adaptive immunity toward host protection against the infection (30, 31).

## Trained Immunity as a Strategy Against SARS-CoV-2

SARS-CoV-2 is an emerging zoonotic virus belonging to the *Coronaviridae* family (32) that was isolated as a result of an outbreak in December 2019, in residents of the Wuhan town, Hubei province, China (33). Since its detection, SARS-CoV-2 has expanded exponentially to different regions of the world, spreading to more than 185 countries and being declared a pandemic on March 11, 2020, by the World Health Organization (WHO). SARS-CoV-2 produces a respiratory syndrome named COVID-19 (Coronavirus disease 2019), which main symptoms include fever above 38°C, dyspnea, shortness of breath, and a dry cough (34). This respiratory disease can trigger pneumonia and even death in more extreme cases (34, 35). One of the biggest problems of the SARS-CoV-2 pandemic is the absence of an effective antiviral treatment or a vaccine, capable of counteracting the inflammatory response and even severe acute damage to the lungs (34). As of April 22, 2020, 2,623,231 SARS-CoV-2 infected people have been reported worldwide, and 133,261 people have died (Center for Systems Science and Engineering, CSSE, Johns Hopkins University). In Chile, the Ministry of Health has reported 11,296 people infected with SARS-CoV-2 and 160 deceased (To see updated data, please follow the next link http://www.imii.cl/en/confirmed-covid-19-cases-per-million-inhabitants/).

**Figure 2:**
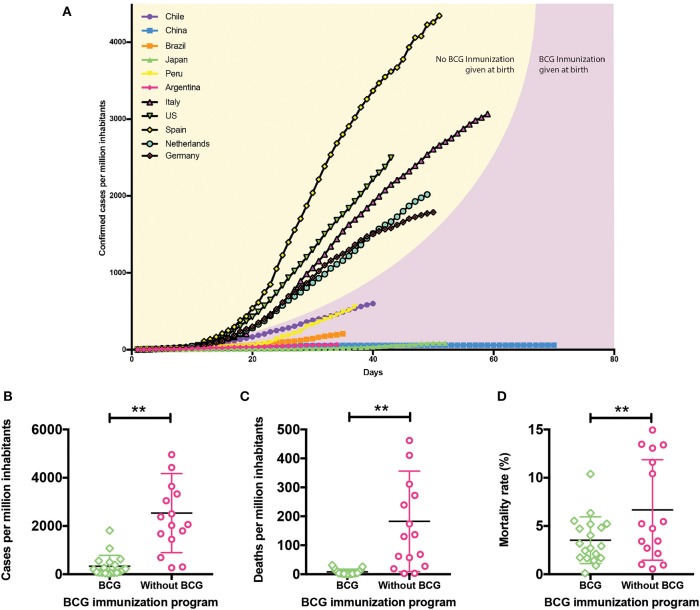
Protective role of BCG in SARS-CoV-2 infection. **(A)** Confirmed cases of COVID-19 since the day they exceeded 2 cases per million up to date. Country curves with a black line and yellow background correspond to those without BCG vaccination program. Country curves with a pink background correspond to those where BCG vaccination is administered at birth. **(B)** Confirmed cases of COVID-19 per million inhabitants, **(C)** deaths per million inhabitants, and **(D)** mortality rates in countries with or without BCG vaccination schedule. Statistical Method: Each group represents the mean ± SD (error bars) of the responses in populations vaccinated (22 countries) and unvaccinated (16 countries) with BCG. Data were compared by *t*-test with a confidence interval of 95% to discriminate statistically significant differences between groups (**), we determine that variances are equals, contrasted by F test (*P* < 0.05). To see updated data, please follow the following link http://www.imii.cl/en/confirmed-covid-19-cases-per-million-inhabitants/ (Source: Center for Systems Science and Engineering, CSSE, Johns Hopkins University, Accessed on April 22, 2020; World Health Organization, WHO).

Based on the information published by the CSSE, we elaborated the graph in Figure 2, which shows the confirmed cases of COVID-19 per million inhabitants in different countries up to date (Table S1). Italy, Spain, and the US show the highest contagion rate, with a sustained increase since the first reported cases. The Netherlands and Germany show a significant increase in their confirmed cases per million inhabitants, suggesting that the contagion curve will increase similar to the Italian, Spanish, and American ones. A common feature of these countries is that they do not include BCG in their national vaccination programs, so we speculate that this vaccine may have a protective role in the immune defense against respiratory diseases (36–41). Utilizing the WHO immunization monitoring data (9), we elaborated the graphs shown in Figures 2B–D comparing the contagion and mortality rates due to COVID-19 between countries with or without administration of BCG in their national vaccination programs. Interestingly, there are significant differences in the confirmed cases per million inhabitants between BCG vaccinated and non-vaccinated countries (Figure 2B). Those countries where BCG is included in their vaccination program have fewer confirmed cases, suggesting that the use of this vaccine may lower the probability of infection. On the other hand, when analyzing death frequencies (Figures 2C,D), those countries without BCG included in their vaccination program exhibit a higher amount of deaths per million inhabitants and higher mortality rates concerning those where BCG vaccination is administered at birth. Interestingly, these data are in accordance with very recent results showing an inverse correlation between BCG vaccination and COVID-19 incidence and mortality (41). These data suggest that BCG vaccination prevents not only SARS-CoV-2 infection but also reduces the probability of developing a severe case of the disease, improving survival rates (41). Since BCG is a specific vaccine against *M. tuberculosis* infection (42), and it has been shown to induce the development of trained immunity (23), these data suggest a crucial role for this vaccine in the development of unspecific memory against respiratory viruses, like SARS-CoV-2. As mentioned above, the “trained” phenotype lasts for a limited time (15), suggesting that trained immunity developed at birth might not be able to protect adults against later infections. However, some studies have shown that neonatal BCG immunization reduces the occurrence of asthma in adolescents reporting rhinitis, suggesting that this non-specific immune effect could be long-lasting (43). Besides, BCG vaccination at birth correlates with a diminished incidence of asthma in adults (26). A study performed in Spain, where BCG vaccination is only administered in the Basque Country, showed that BCG vaccination at birth reduces hospitalizations of children under 14 years of age due to respiratory infections or sepsis (11). Also, severity of COVID-19 cases in Spanish children, who did not receive BCG vaccinations at birth, was significantly higher as compared to Chinese children, with 60 and 2.8% hospitalization rates, respectively (44, 45). These data further support the notion that BCG vaccination at birth may have a long-lasting protective effect. The induction of trained immunity has been described, as mentioned above, for BCG (14, 36), β-glucan (16), or *Candida albicans* (17). Since all of them are pathogens or pathogen components, one can speculate that exposure to different pathogens during a lifetime may strengthen BCG-induced trained immunity at birth, just as revaccination does (46, 47) Thus, it is possible that trained immunity induced by BCG vaccination at birth might have a protective effect against COVID-19. This statement can not be extrapolated to other coronaviruses, like SARS-CoV or MERS; although they are intimately related (32), they are different pathogens to which trained immunity may not have a protective effect.

Among the limitations of our analyses, we are aware that the results presented in Figure 2 may be biased by a wide variety of factors (48). In all cases, diagnosis depends on the amount of testing made in each country. At higher numbers of confirmed cases the mortality rate would be lower, which is the reason why we determined the number of deaths per million inhabitants, since that number is not affected by the number of diagnoses. On the other hand, contagion rates vary depending on the social distancing measures taken by each government. Mortality is also subjected to the demographic distribution of the population of each country. Countries such as Italy or Spain that have higher death frequencies have median ages of 47 and 45 years, respectively, while countries like China or Chile that have lower death frequencies also have younger median ages, being 38 and 35 years old, respectively (49). Other variants that should be considered is the availability of medical treatment and the populational density, among others.

All the variants mentioned before affect the contagion and mortality rates of each country, which is why we can speculate that BCG vaccination may contribute to a difference between the countries, but could not attribute all the differences to it. Nevertheless, BCG vaccination policy correlates with a better tendency toward lower death mortalities and diminished contagion rates. Based on these observations, we hypothesize that BCG vaccination at birth could induce a trained immunity state which could activate a more efficient immune response in SARS-CoV-2 infection.

## Concluding Remarks

Today we live in an age where globalization, population growth, and climate change, combined with zoonotic infections, can threaten public health and our economic and social structure. Given the complex scenario, emerging viruses like SARS-CoV-2 that can cause pandemics pose a real threat for which we are not prepared. That is why vaccines or treatments are urgently required to control or decrease the amounts of contagion and deaths caused by this virus. Trained immunity has been described as an unspecific memory carried by the innate immune system that can provide us with protection against novel infections (7, 8, 23, 30). BCG vaccine has proven its immunogenicity and safety since it has been used for almost a 100 years in humans. Further, trained immunity is induced by this vaccine (23). Along these lines, based on its safety as a vaccine in large populations, BCG could be considered for its broad availability and low cost as a good strategy for the development of trained immunity and, in consequence, protection against novel pathogens in the case of a pandemic. Indeed, two different clinical trials support the idea that BCG revaccination induces a stronger activation of the non-specific cross-protection associated to this vaccine (46). The first one, performed between 1935 and 1947, showed that children's revaccination diminished their overall mortality progressively. The first vaccination reduced it by only 3%, but they achieved a 47% of reduction of mortality in children after the third revaccination (46). Another clinical trial performed in Guinea-Bissau also demonstrated diminished mortality in revaccinated children, with a reduction of 64% (47). These results suggest that revaccination could be able to activate trained immunity in a stronger way as compared to the first induction, thus giving more protection to unrelated pathogens. In this context, BCG revaccination may act as a protective vaccine against COVID-19. Even though the data presented in this article suggest that BCG may have a protective role in infection with SARS-CoV-2, clinical trials in adults might be done to prove this hypothesis. Indeed, currently, the capacity of BCG-induced trained-immunity to protect against COVID-19 is being evaluated in two clinical trials. One is being conducted in Holland, involving 1,500 participants and 147 health care workers who are to be vaccinated, and another in Australia with 4,000 participants and 148 volunteers who will be vaccinated (ClinicalTrials.gov Id: NCT04328441 and NCT04327206, respectively).

## Data Availability Statement

All datasets presented in this study are included in the article/Supplementary Materials.

## Author Contributions

CC, AR-D, and AK wrote the manuscript. SB reviewed the manuscript. AK reviewed and approved the version to be published. All authors listed have made a substantial and intellectual contribution to the work.

## Conflict of Interest

The authors declare that the research was conducted in the absence of any commercial or financial relationships that could be construed as a potential conflict of interest.
