# Supplementary material for: Could BCG Vaccination Induce Protective Trained Immunity for SARS-CoV-2?
Source: Front Immunol. 2020 May 8;11:970. doi: 10.3389/fimmu.2020.00970 (PMC7227382; doi:10.3389/fimmu.2020.00970)
Supplement: Supplementary file 1 [file Table_1.DOCX]

Supplementary Material

| **BCG vaccinated** | **Unvaccinated** |
| --- | --- |
| China | Italy |
| Brazil | US |
| Japan | Spain |
| Chile | Netherlands |
| Peru | Germany |
| Argentina | France |
| Iran | UK |
| Malaysia | Switzerland |
| Poland | Belgium |
| Romania | Austria |
| Pakistan | Australia |
| Thailand | Denmark |
| Saudi Arabia | Luxembourg |
| South Africa | Moldova |
| India | Iceland |
| Singapore | New Zealand |
| Colombia |  |
| Croatia |  |
| Hungary |  |
| Panama |  |
| Hungary |  |
| Iraq |  |

**Table S1.** **Countries included in the analyses shown in figure 2a, b, c.** Selected countries correspond to those with over 1,000 confirmed cases of COVID-19, with a stable vaccination program since 1980. Positive BCG vaccination program was considered when vaccination frequency is over 80%.
